# Comprehensive Analysis of Alternative Polyadenylation Events Associated with the Tumor Immune Microenvironment in Colon Adenocarcinoma

**DOI:** 10.2174/1389202924666230503122134

**Published:** 2023-06-23

**Authors:** Fangning Pang, Peng Yang, Tongfei Wang, Xuzhao Li, Xiaoyong Wu, Rong Yue, Bin Bai, Qingchuan Zhao

**Affiliations:** 1 Department of Surgery, Xi'an International Medical Center Hospital, Xi'an, China;; 2 Institute of Health and Rehabilitation Science, School of Life Science and Technology, Xi'an Jiaotong University, Xi’an, China;; 3 Department of Oncology, Xi’an No. 3 Hospital, Xi’an, China;; 4 Department of Surgery, People's Hospital of Ningxia Hui Autonomous Region, Yinchuan, China;; 5 Department of General Surgery, Affiliated Danzhou People’s Hospital of Hainan Medical University, Danzhou, China;; 6 Department of Emergency, Xi'an Daxing Hospital, Xi'an, China;; 7 Department of Surgery, Xijing Hospital of Digestive Diseases, Fourth Military Medical University, Xi’an, China

**Keywords:** Alternative polyadenylation, COAD, 3'-UTR, immunity, NK cells, T cells

## Abstract

**Objective:**

Colon adenocarcinoma (COAD) is one of the leading causes of cancer death worldwide. Alternative polyadenylation (APA) is relevant to the variability of the 3'-UTR of mRNA. However, the posttranscriptional dysregulation of APA in COAD is poorly understood.

**Methods:**

We collected APA data from The Cancer Genome Atlas (TCGA) COAD (n =7692). APA events were evaluated using PDUI values, and the prognostically significant APA events were screened by LASSO Cox regression to construct a prognostic model. Then, prognostic model functions and possible regulatory genes of characteristic APA events were analyzed. Finally, the immune regulatory network based on APA regulatory genes was analyzed and established.

**Results:**

A total of 95 APA events were found to influence the COAD outcomes. Among them, 39 genes were screened as characteristic prognostic APA events by LASSO Cox regression to construct a COAD prognostic signature. The analysis results suggested that a high signature score was associated with poor prognosis and was significantly correlated with a variety of immune cells, including NK and Th1, 2 and 17 cells. Further analysis showed that APA regulators mainly served roles in the prognosis of COAD. Based on the above results, we constructed an immunoregulatory network for APA regulatory genes-APA genes-immune cells.

**Conclusion:**

Our study revealed that APA events in COAD may regulate tumor progression by influencing immune cells, which provides a new direction for exploring the influencing mechanism of the tumor immune microenvironment and is expected to provide a potential new target for COAD immunotherapy.

## 1 INTRODUCTION

Colon adenocarcinoma (COAD), the main pathological type of colorectal cancer (CRC), is one of the most common malignant tumors globally; the diagnosis rate and the mortality rate represent approximately 1/10 of total cancer cases [1, 2]. In recent years, the incidence and mortality of COAD have increased annually, ranking among the top five of all cancers in China [3]. The incidence of COAD is mainly related to age and eating habits and partly related to genetic diseases [4]. Treatments for COAD mainly include radiation, chemotherapy, surgery targeted therapy, precision therapy and immunotherapy. Currently, immune checkpoint inhibitors (ICIs) have been gradually applied in the clinic and enabled breakthrough progress in the treatment of various malignant tumors [5, 6]. However, immune escape restricts the therapeutic effect of ICIs therapies, and the current management of COAD does not result in favorable remission rates. Alternative polyadenylation (APA) is an important type of posttranscriptional regulation in eukaryotic cells. APA is a widespread phenomenon, generating mRNAs with alternative 3' ends. Approximately 70% of known human genes contain multiple poly(A) sites in the 3' untranslated region (3'-UTR) [7]. APA contributes to the complexity of the transcriptome by regulating the function, stability, translation efficiency and localization of target RNAs [8]. The variability of 3' untranslated regions of mRNA caused by APA affects miRNA–mRNA interactions. During the alteration of polyadenylation sites, a shorter 3'-UTR is generated by choosing the PASs that are most proximal to the translated region. This eliminates miRNA-binding sites and causes the mRNA to lose the suppression effect of miRNA and altered expression [9, 10]. Global 3'-UTR shortening was found to be associated with cell transformation and cancers by eliminating miRNA binding sites of cell growth control factors. While numerous cases of 3'-UTR shortening have been linked to increased protein levels and oncogene activation [11], 3'-UTR shortening may also result in changes in the secondary structure of the mRNA, exposing hidden cis-elements and leading to decreased protein levels [12]. Alternatively, in cases where proximal poly(A) signals are within introns or coding exons, truncated proteins can be generated with potentially different and/or opposing functions [13]. 3'-UTR APA also leads to localization differences within the cytoplasm, as long 3'-UTRs facilitate mRNA localization to the endoplasmic reticulum and accelerate the expression of membrane proteins [14]. Therefore, APA contributes to the oncogenic phenotype through various mechanisms [15]. Recent studies have shown that APA alterations are displayed in a series of biological processes, such as development, cell differentiation, proliferation, neuron activity and cancer [8]. In addition, a large-scale analysis showed that APA events are involved in reshaping cellular pathways and regulating specific gene expression in 17 types of cancer, providing new insights into the pathological mechanism of cancer development [16]. Genes undergoing altered APA during cancer progression may be useful novel biomarkers and potentially targeted for disease prevention and treatment [17]. Previous studies have shown that APA was involved in processes and pathways relevant to CRC tumor biology, such as cell–cell and cell‐matrix interactions, tumor cell growth and metastasis [18, 19]. Zhang *et al*. [20] have examined the relationship between APA and CRC. However, the data and algorithm used by the author have recently become more accurate. In order to understand the relationship between APA and COAD more accurately, we used a new algorithm for analysis. At the same time, in view of the important role of APA regulatory genes in the formation of APA, we conducted a multi-omics analysis of APA regulatory genes. Our analysis revealed that a variety of immune cells were related to APA regulators. An immunoregulatory network for APA regulatory genes-APA genes-immune cells was established which may provide new biomarkers and potential therapeutic targets for COAD immunotherapy.

## 2 MATERIALS AND METHODS

### 2.1 Data Acquisition

RNA-seq, whole genome sequencing (WGS) and clinical parameter data of TCGA-COAD were downloaded using UCSC XENA. APA events in RNA-seq data were evaluated by percentage of distal poly(A) site usage index (PDUI) values. PDUI values represent the frequency of APA events, ranging from 0 to 1, where a larger PDUI correlates with more remote polyadenylate sites using the transcript. PDUI values for all genes of TCGA-COAD per patient were downloaded from TC3A [21] (Supplementary file).

### 2.2 Prognostic Model Construction and Clinical Feature Analysis

Cox regression was used to analyze the influence of APA events on the overall prognosis of COAD in TCGA-COAD. APA events affecting the prognosis of COAD were selected with *p* < 0.05 as the screening criterion. The minimum absolute contraction and selection operator (LASSO) regression model is widely used in the Cox proportional risk regression model for survival analysis of high-dimensional data. To minimize the risk of overfitting, the LASSO Cox regression model (R package “glmnet”) was usually utilized to narrow down the candidate genes and to develop the prognostic model [22]. Therefore, we performed LASSO regression for prognostic APA events. Then, the minimum lambda was used to screen for conforming APA events as characteristic APA events affecting COAD prognosis. Finally, the prognostic model of COAD was constructed based on characteristic APA events. The relationship between the APA prognostic correlation model and COAD characteristics was further analyzed. We further analyzed the relationship between the following TCGA-COAD clinical parameters and the APA prognostic model, including age, sex, chemoradiotherapy, MSI typing and TNM stage. At the same time, the Kaplan‒Meier algorithm was used to analyze the overall survival, progression-free interval (PFI), disease-specific survival (DSS) and disease-free interval (DFI). Finally, the APA prognostic model was divided into two groups with high and low expression according to the optimal grouping of Kaplan-Meier result.

### 2.3 Functional Analysis of the Prognostic Model

Differentially expressed gene profiles were generated with respect to good and poor survival by the “limma” R package. Log2 | fold change | > 1.0 and adjustment *p* < 0.05 were chosen as the standards to screen differentially expressed genes. Furthermore, the possible influence of the prognostic model on function is discussed. First, the gene set enrichment analysis (GSEA) algorithm was used to analyze the Kyoto Encyclopedia of Genes and Genomes (KEGG) function of differential analysis. Second, a gene set variation analysis (GSVA) algorithm was used to evaluate the immune infiltration score and tumor characteristic pathway score of COAD patients, which were used to analyze the effects of good prognosis and poor prognosis on immune cells and tumor characteristic pathways. Finally, the “cluster Profiler” R software package was used for functional enrichment analysis. Enrichment analysis included the Gene Ontology terminology, biological processes, cell composition and molecular function categories, as well as the KEGG pathway. *p* < 0.05 was considered a significant enrichment result.

### 2.4 Analysis of APA Core Regulatory Genes

The GO term stores multiple genes associated with mRNA polyadenylation. APA core regulatory genes are involved in the mRNA polyadenylation of GO: 0006378. The specific expression forms of the above regulatory genes in COAD were analyzed. First, the relationship between APA regulatory genes and COAD occurrence was observed by differential expression analysis. Second, Kaplan‒Meier analysis was used to analyze the influences of regulatory genes on the overall prognosis of COAD. Furthermore, copy number variation data were used to observe the changes in the copy number of regulatory genes. Finally, mutations in regulatory genes in COAD were analyzed.

### 2.5 Study on the Regulatory Mechanism of Characteristic APA Events

Based on the fact that APA regulatory genes perform a regulatory function in APA events, after Spearman correlation analysis was used to observe the relationship between prognostic APA events and regulatory genes, we observed the possible regulatory genes that affect each APA event, in which R2 < 0.25 was used as a screening criterion. Finally, the relationship between APA events and gene expression was further observed because APA events also reflect their own gene expression.

### 2.6 Statistical Analysis

R software V4.1.2 was used for statistical analysis of the data and visualization. The measured data are expressed as the mean ± standard deviation. The Wilcoxon rank sum test was used to estimate differences between two groups. Kaplan‒Meier log rank sum analysis was used to assess survival differences between groups. Cox regression was used to assess the prognostic impact of APA events on COAD. *p* <0.05 was considered statistically significant.

## 3 RESULTS

### 3.1 Establishment of a Characteristic APA Prognostic Model for COAD

**Fig. (2):**
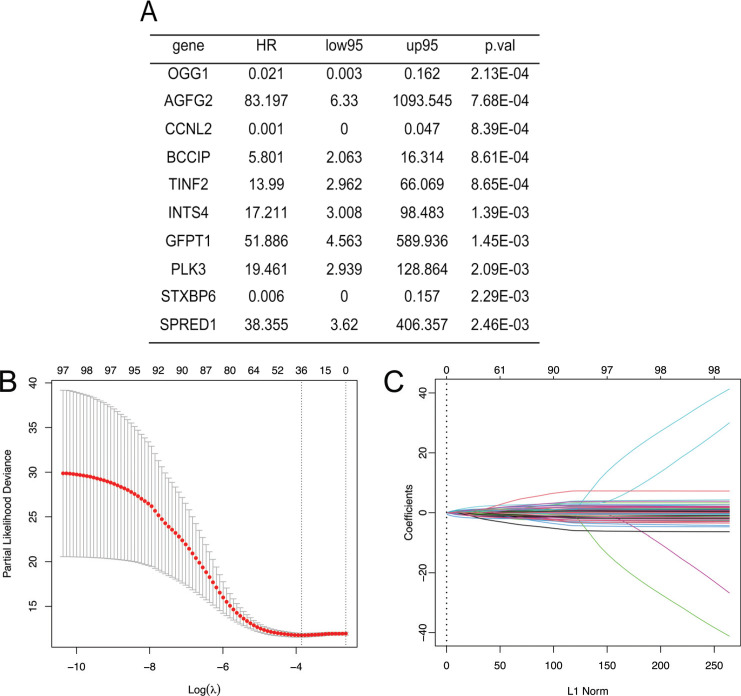
Construction of the prognostic model for APA events. **(A)** The top 10 APA events with prognostic significance. **(B)** The choice of lambda for LASSO regression. **(C)** Characteristic APA event selection for LASSO regression.

As shown in Fig. (**1**), the flow chart demonstrates the overall design framework and analysis process of this study. By analyzing TCGA-COAD data downloaded from TC3A, a total of 7692 APA events were found in COAD. The correlation between APA events and COAD prognosis was analyzed by Cox regression. Analysis revealed that a total of 95 APA events affected the prognosis of COAD (Fig. **2A**). GO analysis was performed on the above 95 genes, and it was found that the above 95 genes are mainly related to the response to oxidative stress, signaling pathway by p53 class mediator and intracellular transport, *etc*. (Supplementary Fig. **1**). Considering the collinearity between genes in univariate Cox regression, LASSO regression was used to analyze the above 95 genes. Candidate genes were screened with 0.01932572 as the minimum lambda value, and a total of 39 genes were ultimately obtained as the characteristic prognostic APA events characteristic of COAD (Fig. **2B** and **C**). KEGG enrichment analysis was performed on the above 39 genes. These genes were found to be mainly related to the combination of the intestinal immune network for IgA production, amino sugar and nucleotide sugar metabolism, base excision repair, and so on (Table **1**).

### 3.2 Relationship between the Characteristic Prognostic Model and Clinical Parameters

**Fig. (3):**
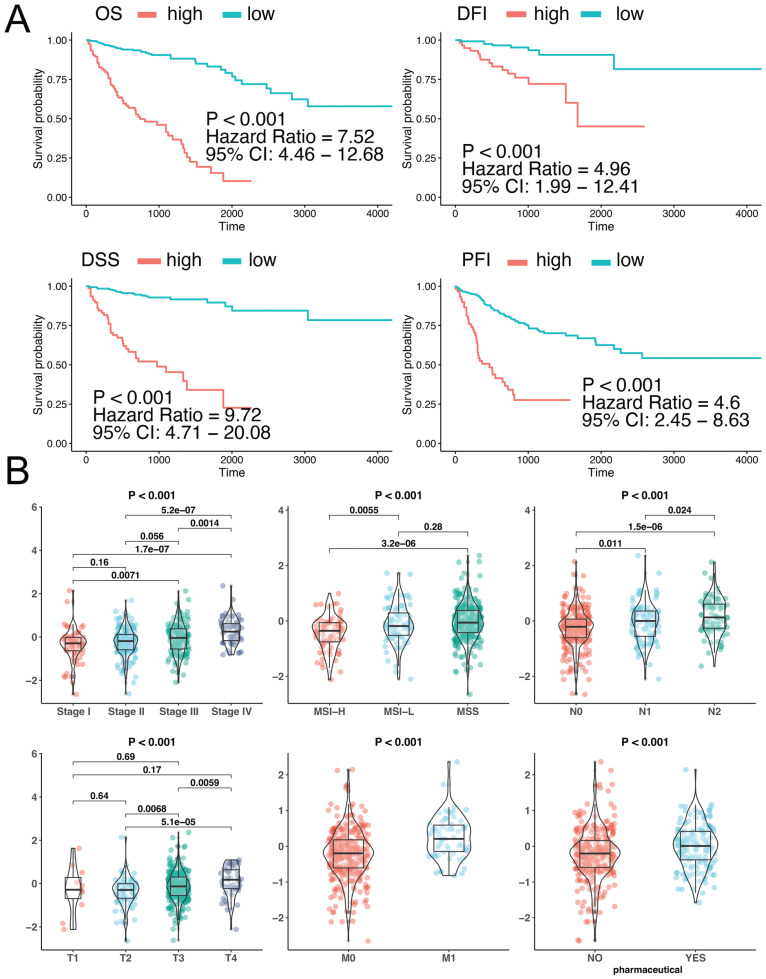
Relationship between the APA signature and clinical features of COAD. **(A)** The influence of the APA signature on the prognosis of COAD. The prognostic analysis of OS, DSS, DFI and PFI is presented from top to bottom. Abbreviations: OS: overall survival; DFI: disease-free interval; PFI: progression-free interval; DSS: disease-specific survival. **(B)** The relationship between the APA signature and COAD clinical information.

Based on the above 39 genes, we fitted a COAD prognostic signature. To explore the basic characteristics of this signature, we analyzed the relationship between COAD clinical parameters and the signature. First, we analyzed the relationship between multiple types of prognostic information of COAD and the APA signature. Analysis showed that the four prognostic indicators were all related to APA events. Patients with high signature scores were associated with poor prognosis, with an HR above 4 (Fig. **3A**). We also analyzed the relationship between COAD clinical features and the signature. Several clinical features were found to be related to the APA signature. STAGE staging data showed that patients with higher STAGE were associated with a higher signature. The same results were presented in the TNM analysis. The above results indicated that a higher signature correlated with more severe disease (Fig. **3B**), which suggests that the characteristic prognostic model may be used as a prognostic indicator. Furthermore, we observed the relationship between treatment and signature. Analysis showed that radiotherapy did not affect the signature, while chemotherapy affected the signature (Supplemental Table **1** and Fig. **3B**).

### 3.3 Functional Analysis of the Prognostic Model

**Fig. (4):**
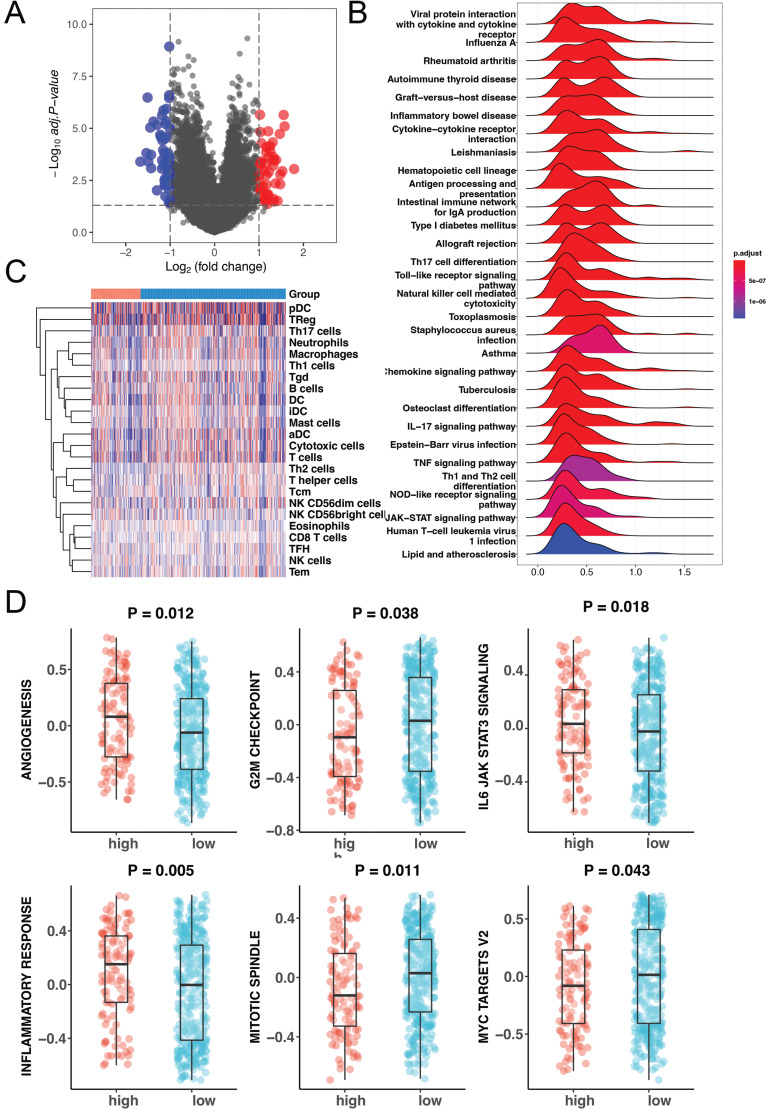
Functional analysis of the APA prognostic model. **(A)** Volcano plot of difference analysis between high and low expression groups. Red: high expression; Blue: low expression. **(B)** GSEA result of difference analysis, where the color represents the specific *p* value and higher-intensity blue color correlates with smaller *p* value. **(C)** Heatmap of immune cells and the APA model. **(D)** Relationship between tumor-related pathways and the APA prognostic model.

We divided the prognostic model into high- and low-score groups based on the optimal grouping of the signature's influence on OS. Based on the above two groups, the possible functional mechanisms were further observed. First, Transcript per million (TPM) data from TCGA-COAD of the two groups was analyzed to detect the differentially expressed genes between the two groups. A total of 39 high-expression genes and 66 low-expression genes were found (Fig. **4A**). To observe the functions of differentially expressed genes, GSEA was performed to interpret gene expression data. There were 108 pathways associated with the prognostic model, including the IL−17 signaling pathway, natural killer cell-mediated cytotoxicity and Th1 and Th2 cell differentiation (Fig. **4B**). To better understand the relationship between the prognostic model and immune cells, we evaluated the immune score of each COAD patient using the GSVA algorithm. By observing the relationship between the immune score and the prognostic model, we found that a variety of immune cells, including NK cells, Th1 cells and Th2 cells, were related to the APA prognostic model (Fig. **4C** and Table **2**). Furthermore, we analyzed the relationship between the prognostic model and 50 classic tumor-related pathways. Analysis showed that the prognostic model indicated changes in only 6 pathways, including angiogenesis, the G2/M checkpoint, and inflammatory cells (Fig. **4D**). These results suggest that the APA prognostic model perform functions mainly by influencing immune cells.

### 3.4 Comprehensive Analysis of APA Regulatory Genes

**Fig. (5):**
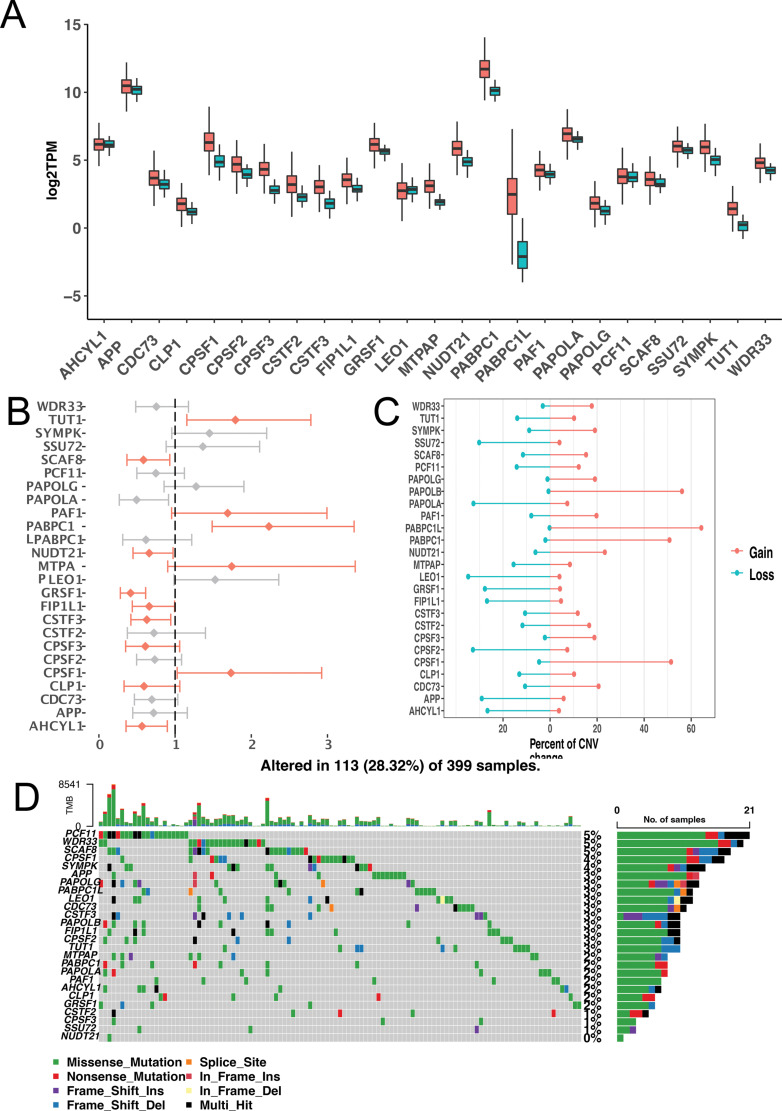
Overall landscape of APA regulatory genes. **(A)** Differential expression of APA regulatory genes in COAD (cancer *vs*. normal). **(B)** Relationship between APA regulatory genes and prognosis of COAD. Red: significant differences; gray: no significant differences. **(C)** Relationship between APA regulatory genes and COAD copy number variation. Red represents an increase in copy number, and blue represents a decline in copy number. **(D)** Analysis of mutations in APA regulatory genes.

To explore the regulatory factors of the APA prognostic model, we conducted a comprehensive analysis of APA regulatory genes. First, 34 polyadenylated regulatory genes were obtained by using GO: 0006378 from the gene GO database as APA core regulatory genes. We further observed the basic changes in these regulatory genes in COAD. Differential expression analysis was performed to determine whether regulatory genes affected the occurrence of COAD. Analysis showed that although multiple genes were highly expressed in COAD, only PABPC1L was differentially expressed (Fig. **5A**). However, in the prognostic analysis, a total of 13 regulatory genes were found to affect the prognosis of COAD (Fig. **5B**). These results suggest that APA regulatory genes mainly impact the prognosis of COAD. Furthermore, based on copy number analysis, it was found that regulatory genes exhibited different copy number changes, among which PABPC1L and CPSF1 mainly showed an increase in copy number, while LEO1 and CPSF2 mainly showed a decrease in copy number (Fig. **5C**). We also found that there were few mutations in regulatory genes, and the most mutated PCF11 gene was mutated in only 5% of the samples (Fig. **5D**). Since the above results suggested that APA events were related to immune invasion by colon cancer, we further analyzed the relationship between APA regulatory genes and immune invasion and found that a variety of immune cells, including NK cells, CD8^+^ T cells and Th2 cells, were associated with APA regulatory genes (Supplementary Table **2**).

### 3.5 Analysis of Core Regulators of APA Events in COAD

**Fig. (6):**
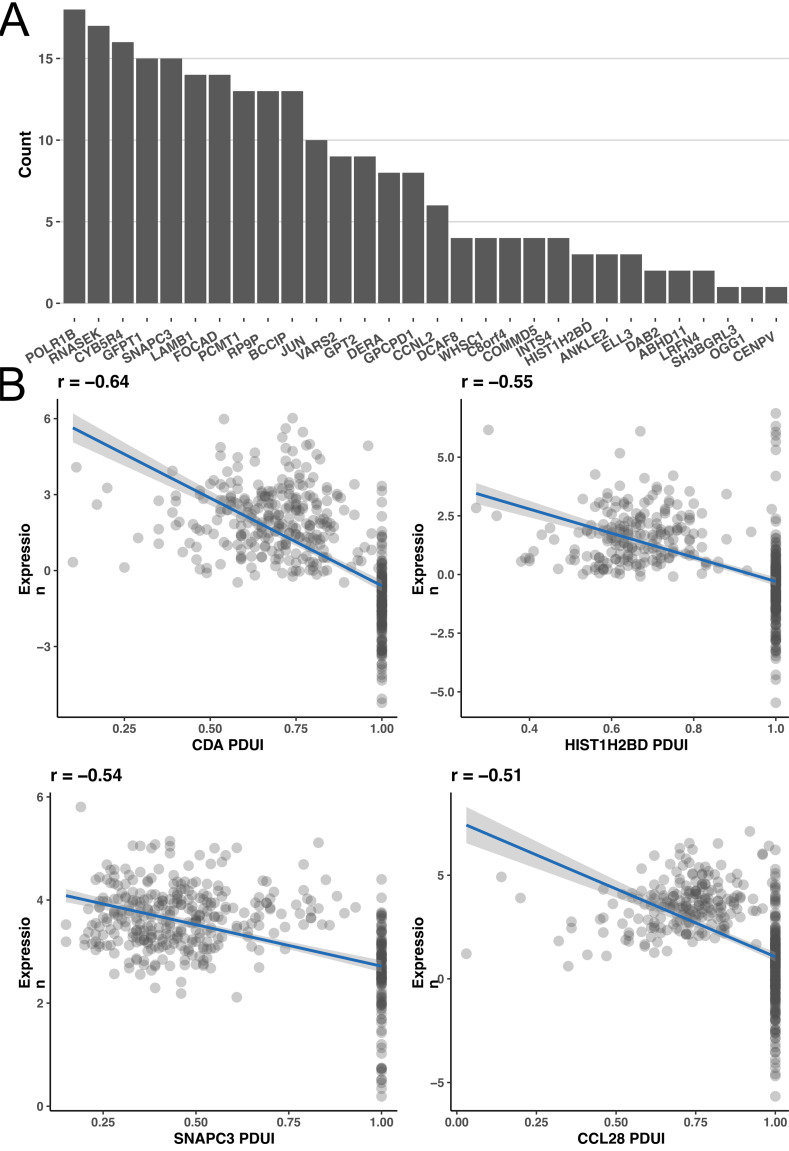
Potential regulatory mechanisms of the APA prognostic model. **(A)** Analysis of COAD characteristic APA events regulated by regulatory genes. **(B)** The influences of APA events on gene expression.

We further analyzed the potential regulatory relationship between each characteristic APA event and regulatory gene. Analysis revealed that some events were positively regulated by APA regulatory genes, such as ABDH11, while others were negatively regulated, such as RNASEK (Fig. **6A**). In addition to the regulated APA events, there were nine APA events that had no relationship with regulatory genes. These results indicated that the currently known regulatory genes may be incomplete, and the regulatory mechanism of APA events requires further exploration. We also observed the relationship between APA events and their gene expression and found that 24 genes were negatively correlated in this regard, among which the negative correlation of the CDA gene reached -0.6 (Fig. **6B**).

### 3.6 Immunoregulatory Network Construction for APA Regulatory Genes-APA Genes-immune Cells

**Fig. (7):**
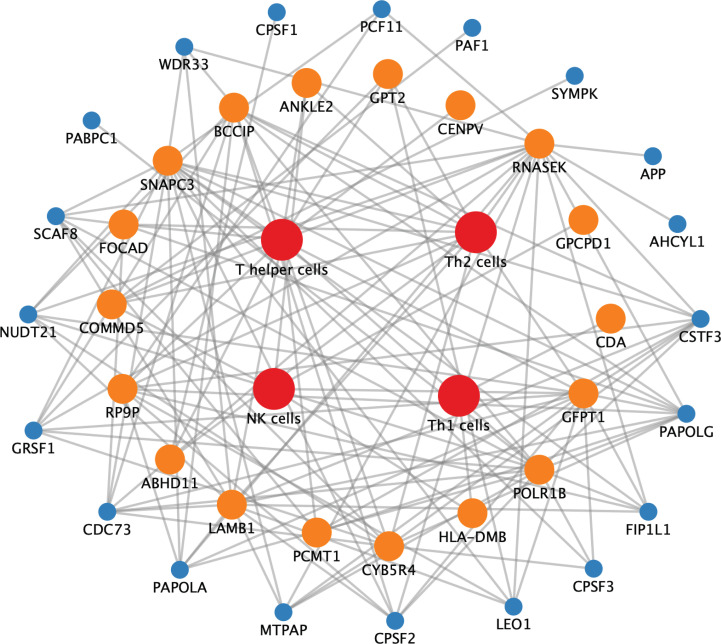
APA regulatory genes-APA genes-immune cells regulatory network. Blue nodes: APA regulatory genes; Green nodes: APA genes; Orange nodes: immune cells.

Since immune cells in COAD tumor tissues are affected by both APA events and APA regulatory genes, we conducted network construction to obtain a better understanding of the possible regulatory relationships between them. After analysis, we found that a variety of immune cells interact with APA events and APA regulatory genes. Th2 and NK cells were most affected by APA genes and APA regulatory genes (Fig. **7**).

## 4 DISCUSSION

COAD is one of the most prevalent malignant tumors worldwide and has a poor prognosis. It is generally accepted that the identification of new diagnostic, predictive and prognostic biomarkers and the development of new treatments are effective means of reducing COAD mortality. To date, most studies on the identification of COAD biomarkers have mainly focused on known molecular pathways and genomic aberrations in the pathogenesis of COAD, such as mutations, methylation changes, and DNA copy number changes. However, the study of new processes that lead to gene dysregulation during carcinogenesis can lead to the discovery of novel biomarkers and therapeutic targets. Recently, an increasing number of studies have suggested that APA is a key event in gene regulation and is highly correlated with cancer progression. APA is derived from the existence of multiple PASs within the same transcript, resulting in the differential inclusion of sequence information at the 3' end and altered posttranscriptional regulation of gene expression. Through alteration of 3'-UTRs, APA potentially regulates the stability, cellular localization and translation efficiency of target RNAs, as 3'-UTRs serve as major docking platforms for factors that control these regulatory layers, such as miRNAs and RNA-binding proteins [23]. APA is considered a fundamental mediator of gene expression and is crucials in many biological processes and diseases, such as cell differentiation, proliferation and cancer [24]. Malignant transformation involves processes orchestrating gene regulation at the posttranscriptional level, often through the 3'-UTRs of mRNAs. Previous studies revealed that APA events add strong prognostic power beyond standard clinical and molecular parameters in the context of several types of cancer, suggesting their potential as novel prognostic biomarkers [10]. Additionally, APA patterns have the potential to be used as diagnostic tools for distinguishing tumor types. In particular, specific patterns of alternative cleavage and APA, leading to alterations in 3'-UTR length and content, have been shown to effectively discriminate between histologically indistinguishable tumors. Changing APA patterns have been shown to result in dysregulation of genes involved in many types of cancers, including colorectal cancer [8]. However, the association between APA and COAD prognosis remains to be fully elucidated. Therefore, we extracted data from TCGA databases to investigate the effects of APA events on colon cancer and explore specific mechanisms. The correlation between APA events and COAD prognosis was analyzed by Cox regression to screen out prognostically significant APA events. To avoid collinearity, LASSO regression was further used to detect characteristic prognostic APA events and to construct a prognostic model. Several clinical features were found to be related to the APA signature. Among them, a high signature score of prognostic information for overall survival, disease-free interval (DFI), progression-free interval (PFI) and disease-specific survival (DSS) was associated with poor prognosis. The TNM stage was positively correlated with the signature score, suggesting that the characteristic APA prognostic model can predict the survival prognosis of COAD patients. Furthermore, we observed the relationship between treatment and the APA signature. In our study, medication was associated with higher signature scores, suggesting that medication may promote the occurrence of APA events, which may be involved in the mechanisms of drug tolerance. Based on the above analysis data, we found that a low signature score indicated a relatively favorable prognosis in COAD patients. Therefore, the prognostic value of the APA prognostic model was preliminarily proven by our results. To reveal the potential causes of APA events affecting COAD prognosis, we divided the prognostic model into two groups with high and low scores based on the optimal grouping of signatures affecting OS and analyzed the DEGs. Further analysis revealed that multiple immune-related pathways are associated with prognostic models, including the IL−17 signaling pathway, NK-mediated cytotoxicity, the toll-like receptor signaling pathway and Th1 and Th2 cell differentiation. Moreover, a comparison of the immune scores of patients with poor prognosis and those with good prognosis revealed significant differences in immune infiltration between the two groups, especially in NK cells and Th1, Th2 and Th17 cells, suggesting that APA may exert influence prognosis mainly by affecting immune cells. The immune system exerts influences and effects in the occurrence and development of cancer [25]. Tumor-infiltrating lymphocytes (TILs), part of the host immune response, have been widely reported as influential factors in determining the outcomes of patients with colon cancer. Studies have shown that the presence of highly infiltrated TILs in tumors, especially CD^4+^ T cells and CD^8+^ T cells, is related to the good clinical prognosis of patients, which is manifested by longer PFS and OS [26]. T-helper 1-cell-mediated responses against established colorectal tumors are associated with better outcomes of patients, whereas Th2 cells may be antagonists of CTL induction, in contrast to Th1 cells [27]. In CRC, it has been reported that Th1-cell predominance is correlated with good prognosis, whereas a high proportion of Th2 cells is correlated with poor prognosis [28]. Th17-cell-mediated responses and the production of interleukin 17a have been associated with worse patient outcomes. Indeed, tumor-infiltrating Th17 cells have been found in various human cancers, confirming their protumorigenic properties [29]. Additionally, NK cells have been described to eliminate tumors and metastases and thus to be pivotal in innate and adaptive immune responses and tumor immune surveillance [30]. They have been associated with increased survival of colon cancer patients [31]. Apart from their cytotoxic function, NK cells also produce cytokines and chemokines that regulate other immune cells, such as recruiting dendritic cells (DCs) through secretion of factors such as CCL5, XCL1, XCL2 and IFN-γ, the latter enhancing DC-induced Th1 polarization [32]. In addition, we noticed that some immune cell subsets such as Tem, Tfh and macrophages were not significantly associated with APA prognostic models. Due to the complexity of the tumor immune microenvironment, these subsets may be regulated by other mechanisms, which require more in-depth experiments to explore. To further clarify the undisclosed mechanism by which APA events affect immune cells, we conducted a comprehensive analysis of potential APA regulatory genes. Consistent with our expectations, the analysis revealed that APA regulatory genes exert their influence mainly by influencing COAD prognosis. Moreover, a variety of immune cells, including NK cells, CD^8+^ T cells, Th2 cells and Th1 cells, are related to APA regulators. Indeed, there are already a few well-documented examples of the robust role of APA in regulating apoptosis in the immune system by controlling the expression of several apoptosis regulators [33]. APA regulates the behavior of the immune system, and control of APA in immune cells has been observed to be driven both by changes in the expression of core CPAFs and *via* the activity of specific RNA binding proteins (RBPs). Although proteomics methods have identified over 80 proteins that may be involved in mRNA 3' end processing, the functions of most of these factors in APA remain unclear [34]. The regulation of gene expression in immune cells is highly complex; however, our understanding of the mechanisms and effects of APA regulation of immune cells is still relatively limited. To further reveal the mechanism by which APA regulatory genes regulate immune cells, we constructed an immunoregulatory network for APA regulatory genes-APA genes-immune cells. Currently, novel therapies, such as immune checkpoint inhibitors (ICIs), have enabled breakthrough progress in the treatment of various malignant tumors [35]. However, immune escape restricts the therapeutic effect of these therapies, and the current management of COAD cannot achieve favorable remission; a large proportion of patients do not obtain long-lasting clinical effects [36]. Improving the therapeutic effectiveness of these patients who do not benefit from immunotherapy has become a concern of clinicians. Our results outline an immune regulatory network that influences COAD prognosis through APA, and these genes can serve as biomarkers and may be used for potential immunotherapy.

## CONCLUSION

In summary, the novelty of this study lies in the discovery that APA events can affect the immune microenvironment and prognosis of COAD. Furthermore, we constructed an immune regulatory network including APA regulatory genes-APA genes-immune cells, which may provide clues for further mechanistic exploration of immune escape and potential new targets for COAD immunotherapy.

## Figures and Tables

**Fig. (1):**
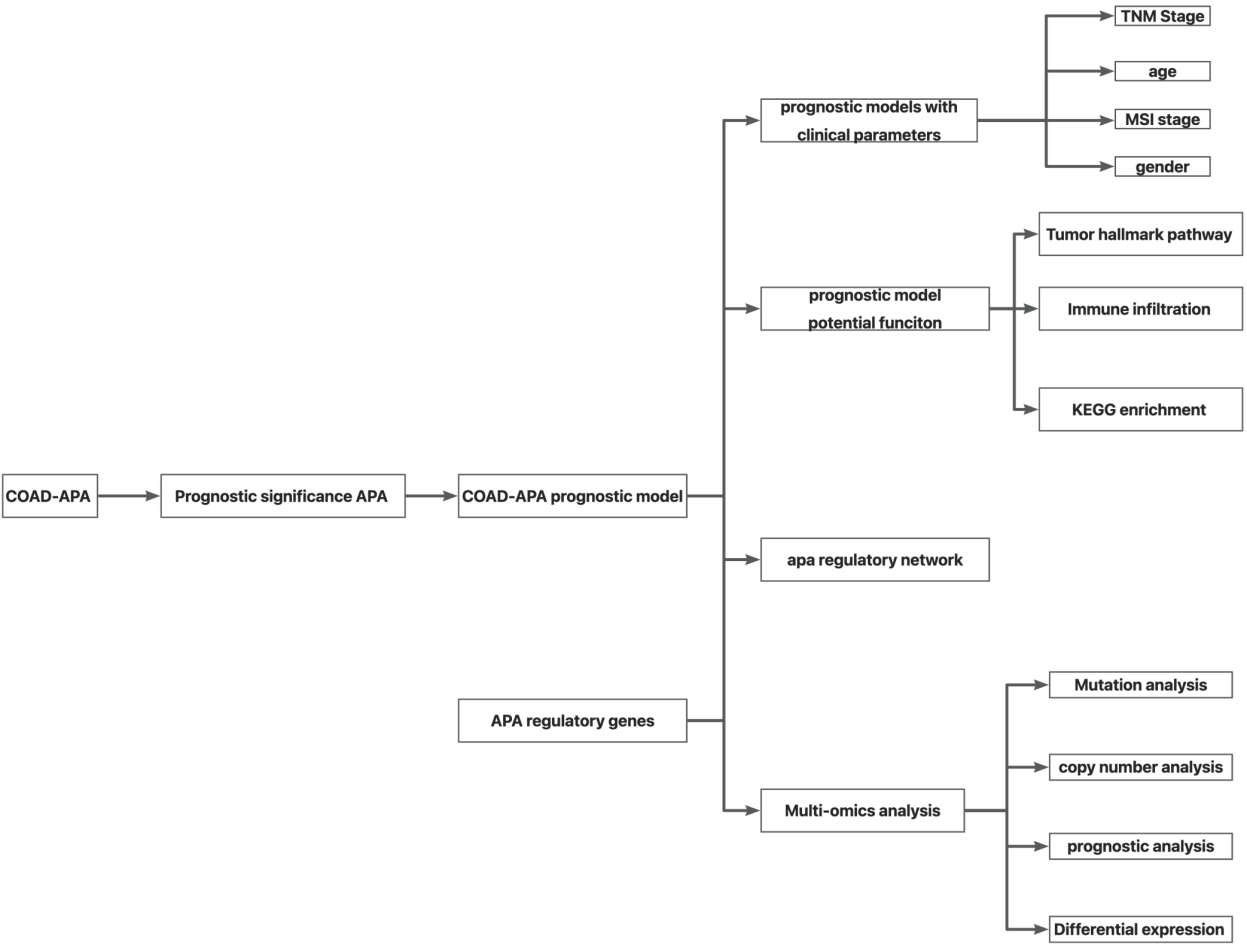
Flow chart of overall study design framework and analysis process. APA data from The Cancer Genome Atlas (TCGA) COAD (n =7692) was collected. APA events were evaluated using PDUI values, and the prognostically significant APA events were screened by LASSO Cox regression to construct a prognostic model. Then, prognostic model functions and possible regulatory genes of characteristic APA events were analyzed. Finally, the immune regulatory network based on APA regulatory genes was analyzed and established.

**Table 1:** KEGG analysis results of characteristic APA genes after LASSO regression screening.

**ID**	**Description**	**GeneRatio**	***p*-value**	**geneID**
hsa03410	Base excision repair	2/13	0.00122289	PARP1/OGG1
hsa00250	Alanine, aspartate and glutamate metabolism	2/13	0.00153693	GFPT1/GPT2
hsa00520	Amino sugar and nucleotide sugar metabolism	2/13	0.00257728	GFPT1/CYB5R4
hsa04672	Intestinal immune network for IgA production	2/13	0.00268452	CCL28/HLA-DMB
hsa05145	Toxoplasmosis	2/13	0.0134031	HLA-DMB/LAMB1

**Table 2:** Analysis of the relationship between the APA prognostic model and immune cells.

**Characteristic**	**High, N = 112^1^**	**Low, N = 328^1^**	***p*-value^2^**
aDC	-0.15 (0.49)	0.01 (0.50)	0.003
B cells	-0.03 (0.42)	0.00 (0.42)	0.67
CD8 T cells	-0.06 (0.24)	-0.01 (0.25)	0.047
DC	-0.08 (0.50)	0.01 (0.48)	0.10
Eosinophils	-0.05 (0.25)	-0.02 (0.26)	0.39
iDC	-0.06 (0.35)	0.00 (0.37)	0.17
Macrophages	-0.08 (0.43)	0.01 (0.43)	0.055
Mast cells	-0.08 (0.38)	0.01 (0.41)	0.026
Neutrophils	-0.13 (0.46)	0.03 (0.46)	0.004
NK cells	-0.11 (0.33)	-0.03 (0.29)	<0.001
pDC	-0.05 (0.66)	0.03 (0.69)	0.21
T cells	-0.12 (0.51)	0.03 (0.51)	0.012
Tcm	-0.06 (0.34)	0.00 (0.34)	0.12
Tem	-0.06 (0.34)	-0.01 (0.34)	0.18
TFH	-0.03 (0.30)	-0.02 (0.29)	0.66
Tgd	-0.05 (0.50)	-0.01 (0.48)	0.38
Th1 cells	-0.11 (0.34)	0.01 (0.34)	<0.001
Th17 cells	-0.16 (0.48)	0.03 (0.47)	<0.001
Th2 cells	-0.18 (0.28)	0.04 (0.33)	<0.001
Treg	-0.10 (0.68)	0.05 (0.68)	0.082

Note: ^1^Mean (SD)

^2^Wilcoxon rank sum test

## Data Availability

The authors confirm that the data supporting the findings of this research are available within the article.

## LIST OF ABBREVIATIONS

3'-UTR: 3' Untranslated Region
APA: Alternative Polyadenylation
COAD: Colon Adenocarcinoma
CRC: Colorectal Cancer
DFI: Disease-Free Interval
DSS: Disease-Specific Survival
GSEA: Gene Set Enrichment Analysis
GSVA: Gene Set Variation Analysis
KEGG: Kyoto Encyclopedia of Genes and Genomes
PASs: Polyadenylation Sites
PDUI: Poly(A) Site Usage Index
PFI: Progression-Free Interval
TCGA: The Cancer Genome Atlas
WGS: Whole Genome Sequencing
