# Supplementary material for: Comprehensive Analysis of Alternative Polyadenylation Events Associated with the Tumor Immune Microenvironment in Colon Adenocarcinoma
Source: Curr Genomics. 2023 Jun 23;24(1):48–61. doi: 10.2174/1389202924666230503122134 (PMC10334702; doi:10.2174/1389202924666230503122134)
Supplement: Supplementary file 1 [file CG-24-48_SD1.zip › CG-24-48_SD1/5b-Supplementary Material.pdf]

Supplementary Material

Comprehensive Analysis of Alternative Polyadenylation Events Associated with the Tumor Immune Microenvironment in Colon Adenocarcinoma

Fangning Pang[1,2,#], Peng Yang[2,#], Tongfei Wang[3,#], Xuzhao Li[4], Xiaoyong Wu[5], Rong Yue[6], Bin Bai[7,*] and Qingchuan Zhao[7,*]

[1]Department of Surgery, Xi'an International Medical Center Hospital, Xi'an, China; [2]Institute of Health and Rehabilitation Science, School of Life Science and Technology, Xi'an Jiaotong University, Xi'an, China; [3]Department of Oncology, Xi'an No. 3 Hospital, Xi'an, China; [4]Department of Surgery, People's Hospital of Ningxia Hui Autonomous Region, Yinchuan, China; [5]Department of General Surgery, Affiliated Danzhou People's Hospital of Hainan Medical University, Danzhou, China; [6]Department of Emergency, Xi'an Daxing Hospital, Xi'an, China; [7]Department of Surgery, Xijing Hospital of Digestive Diseases, Fourth Military Medical University, Xi'an, China

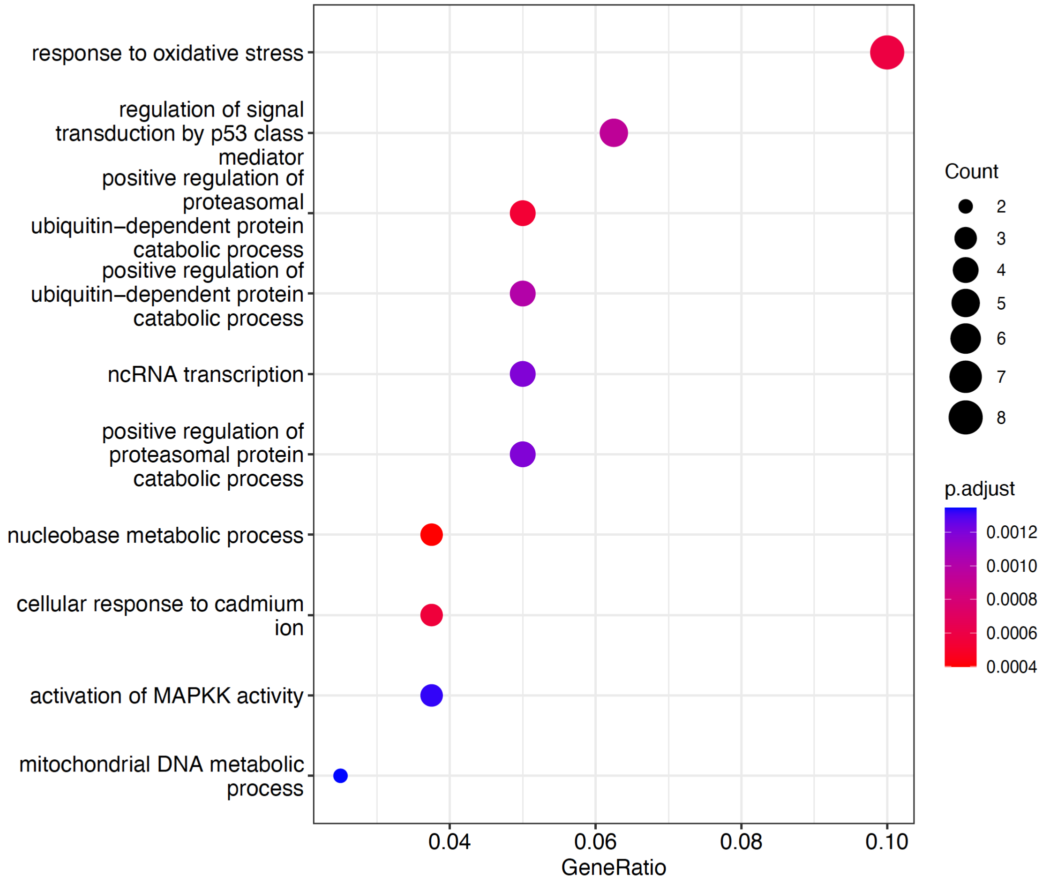

Suppmentary Figure 1. GO analysis results of APA events affecting the prognosis of COAD. Among them, the color of the bubbles represents the P value, and the size of the bubbles represents the number of genes in the enrichment result.

Supplementary Table 1. Influence of the APA prognostic model on radiotherapy.

Characteristic	NO, N = 4291	YES, N = 101	p-value2
lasso	-0.26 (0.64)	-0.20 (0.47)	0.65

1Mean (SD)

2Wilcoxon rank sum test

Supplementary Table 2. Influence.

Gene	Cells	R
CSTF3	CD8 T cells	0.332485
PAPOLG	CD8 T cells	0.37470893
FIP1L1	CD8 T cells	0.31107866
LEO1	CD8 T cells	0.37971698
SCAF8	CD8 T cells	0.35011554
CPSF2	CD8 T cells	0.35582793
PAPOLA	CD8 T cells	0.35163148
CLP1	CD8 T cells	0.30802468
CDC73	CD8 T cells	0.41027561
GRSF1	CD8 T cells	0.31262386
NUDT21	CD8 T cells	0.36530781
WDR33	CD8 T cells	0.30357903
TUT1	Neutrophils	-0.3030708
APP	NK cells	0.3269678
PAF1	NK cells	0.4466753
SYMPK	NK cells	0.30368757
PABPC1L	Th2 cells	-0.3421262
CSTF3	Th2 cells	0.50145014
PAPOLG	Th2 cells	0.41635682
FIP1L1	Th2 cells	0.38931301
CPSF3	Th2 cells	0.35343654
LEO1	Th2 cells	0.56864801
SCAF8	Th2 cells	0.37022783
CPSF2	Th2 cells	0.59378623
MTPAP	Th2 cells	0.46180355
AHCYL1	Th2 cells	0.54383632
PAPOLA	Th2 cells	0.50589115
CDC73	Th2 cells	0.54371106
GRSF1	Th2 cells	0.5976304
NUDT21	Th2 cells	0.49435059
WDR33	Th2 cells	0.33498273
